# Secondary Sulfonamides as Effective Lactoperoxidase Inhibitors

**DOI:** 10.3390/molecules22060793

**Published:** 2017-05-24

**Authors:** Zeynep Köksal, Ramazan Kalin, Yasemin Camadan, Hande Usanmaz, Züleyha Almaz, İlhami Gülçin, Taner Gokcen, Ahmet Ceyhan Gören, Hasan Ozdemir

**Affiliations:** 1 Department of Chemistry, Faculty of Sciences, İstanbul Medeniyet University, 34730 İstanbul, Turkey; zeynep.koksal@medeniyet.edu.tr; 2 Department of Chemistry, Faculty of Science, Ataturk University, 25240 Erzurum, Turkey; rkalin@atauni.edu.tr (R.K.); hozdemir@atauni.edu.tr (H.O.); 3 Department of Basic Science, Faculty of Science, Erzurum Technical University, 25240 Erzurum, Turkey; 4 Pharmacy Services Program, Vocational School of Health Services, Artvin Coruh University, 08000 Artvin, Turkey; yasem_can@hotmail.com; 5 Department of Bioengineering, Faculty of Engineering and Architecture, Sinop University, 57000 Sinop, Turkey; husanmaz@sinop.edu.tr; 6 Department of Molecular Biology and Genetics, Faculty of Sciences and Arts, Muş Alparslan University, 49250 Muş, Turkey; z.turkoglu@alparslan.edu.tr; 7 TUBITAK UME, Chemistry Group Laboratories, P.O. Box: 54, 41470 Gebze Kocaeli, Turkey; taner.gokcen@tubitak.gov.tr (T.G.); ahmet.goren@acgpubs.org (A.C.G.); 8 Department of Organic Chemistry, Faculty of Science, Istanbul Technical University, 34469 Istanbul, Turkey

**Keywords:** lactoperoxidase, secondary sulfonamide, enzyme purification, enzyme inhibition

## Abstract

Secondary sulfonamides (**4a**–**8h**) incorporating acetoxybenzamide, triacetoxybenzamide, hydroxybenzamide, and trihydroxybenzamide and possessing thiazole, pyrimidine, pyridine, isoxazole and thiadiazole groups were synthesized. Lactoperoxidase (LPO, E.C.1.11.1.7), as a natural antibacterial agent, is a peroxidase enzyme secreted from salivary, mammary, and other mucosal glands. In the present study, the in vitro inhibitory effects of some secondary sulfonamide derivatives (**4a**–**8h**) were examined against LPO. The obtained results reveal that secondary sulfonamide derivatives (**4a**–**8h**) are effective LPO inhibitors. The K_i_ values of secondary sulfonamide derivatives (**4a**–**8h**) were found in the range of 1.096 × 10^−3^ to 1203.83 µM against LPO. However, the most effective inhibition was found for *N*-(sulfathiazole)-3,4,5-triacetoxybenzamide (**6a**), with K_i_ values of 1.096 × 10^−3^ ± 0.471 × 10^−3^ µM as non-competitive inhibition.

## 1. Introduction

Sulfonamides constitute a privileged class among pharmacological agents by possessing properties including carbonic anhydrase enzyme (CA) inhibition, as well as diuretic, hypoglycemic, anticancer, antibacterial, antiviral, and metalloprotease inhibitory effects. Although many years passed since their first discovery in the 1930s as a chemotherapeutic agent for antibacterial properties they are receiving increasing attention due to their newly-discovered pharmacological properties [1,2,3,4,5]. Due to the belief that sulfonamides must be be primary sulfonamides to exhibit those pharmacological properties, secondary sulfonamides are more investigated from the synthetic view and have found less pharmacological applications. However, recent investigations shown that secondary sulfonamides have a great potential not only for their selective inhibition characteristics on CA isozymes [6,7,8] but also for their good inhibition properties over cancer-related CA isoenzymes [9], for anticandidal properties [10] and also for their glutamate carboxypeptidase II inhibition properties [11].

It was also reported that sulfonamides had a number of interesting functionalities. Some example can be given for secondary and tertiary and sulfonylureas represent particularly important structural motifs in several classes of drugs. Sulfonamides possessed some biological activities as antibiotics (sulfhamethoxazole), PDE5 inhibitors for the treatment of erectile dysfunction (sildenafil), protease inhibitor for treatment of HIV (darunavir), sulfonylureas for treatment of diabetes mellitus (glibenclamide), hepatitis C anti-viral RNA polymerase inhibitors, and non-steroidal anti-inflammatory COX-2 inhibitors (oxicam class) [12].

Because secondary sulfonamides have been reported to be effective agents, in this study, new designs of secondary sulfonamide drugs containing thiazole, pyrimidine, pyridine, isoxazole and thiadiazole moieties were utilized to obtain acetoxybenzamide, triacetoxybenzamide, hydroxybenzamide and trihydroxybenzamide sulfonamide derivatives to achieve the synergistic effects of sulfonamides and polyphenols in one molecule. Since acetyl protection of hydroxyl groups is a well-known application in pharmacology to enhance oral bioavailability and to increase therapeutic concentration of the drug in the bloodstream, both acetylated and non-acetylated forms of compounds were examined together.

Milk contains a variety of constituents that protect the neonate and the milk itself from a host of deleterious microorganisms. One of the constituents is the lactoperoxidase (LPO) system [13,14,15]. This system is a naturally-occurring antimicrobial system [16,17,18] which is inherently available in raw milk and human body fluids such as saliva. There are three primary components in the LPO system: haeme-containing LPO, hydrogen peroxide (H_2_O_2_), and thiocyanate (SCN^−^). H_2_O_2_ is produced by a number of microorganisms such as lactobacilli, Lactococcus and streptococci through enzymatic oxidation of some biomolecules including ascorbic acid and glucose [19,20,21]. Additionally, LPO is released from mucosal glands and can be found in secretions like saliva, milk or tears. The potential of LPO to inhibit bacterial growth in milk has been recognized [22,23]. LPO catalyses the H_2_O_2_-dependent oxidation of thiocyanate (SCN^−^) to hypothiocyanite (OSCN^−^). The latter ion is a potent antimicrobial agent against gram-negative and gram-positive bacteria, fungi, and viruses [16,24]. This reaction makes the LPO system potentially useful in improving food safety [16,25]. LPO has crucial applications in various fields. For example, LPO protects the intestinal tract system of newborn infants against pathogenic microorganisms by catalysing halides and pseudohalides. LPO is one of the important proteins in bovine whey, and it has been known to play a key role in protection of the lactating mammary gland and the intestinal tract of newborn infants against pathogenic microorganisms [26,27,28].

The aim of the present study was to assess the inhibition effects of a new class of secondary sulfonamides against LPO enzyme, one of the prominent enzymes generally found in several sources, such as bovine milk, saliva, and tears.

## 2. Materials and Methods

### 2.1. Chemicals and Materials

Fresh bovine milk was obtained from the local dairy. L-Tyrosine, Amberlite CG-50-NH_4_^+^ resin, CNBr-activated-Sepharose 4B, protein assay reagent sulfanilamide, and chemicals for electrophoresis were purchased from Sigma-Aldrich Co. (Sigma-Aldrich Chemie GmbH Export Department Eschenstrasse 5, 82024 Taufkirchen, Germany).

### 2.2. General Procedure for Sulfonamide Derivatives

**Figure 1:**
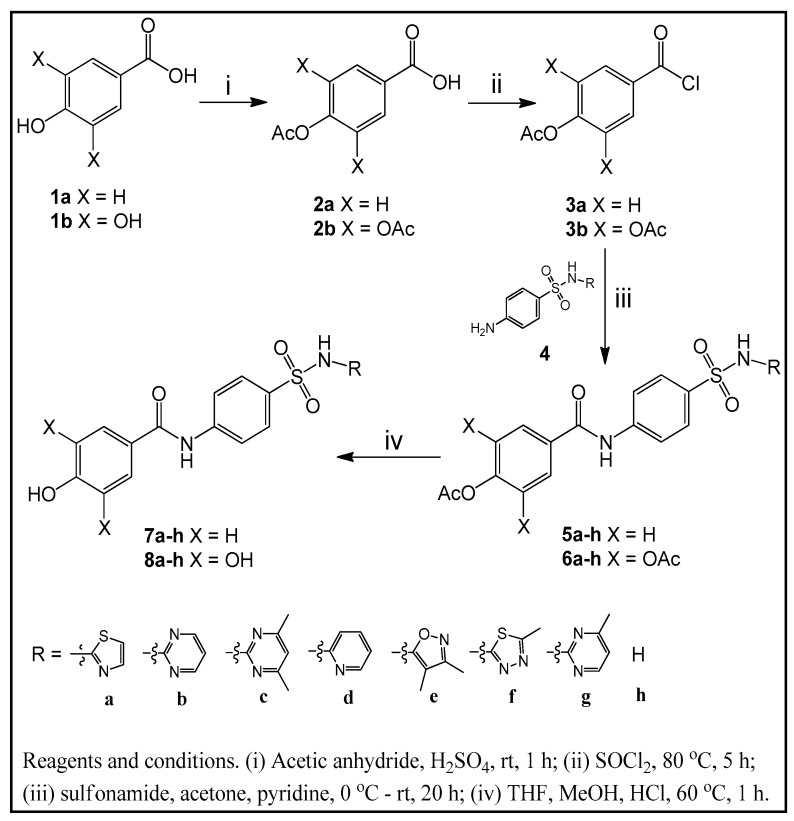
The synthesis route of targeted secondary sulfonamide derivatives (**1a**–**8h**).

Secondary sulfonamides were synthesized using naturally available 4-hydroxy and 3,4,5-trihydroxy benzoic acids (gallic acid) in various plants and fruits, which were reacted with acetic anhydride to obtain 4-acetoxy and 3,4,5-triacetoxy benzoic acids for the protection of hydroxyl groups. Then, they were converted to their corresponding chloride derivatives by treating with thionyl chloride to produce benzoyl chlorides, which underwent reactions with secondary sulfonamides having thiazole, pyrimidine, pyridine, isoxazole and thiadiazole groups to obtain secondary sulfonamide derivatives of acetoxybenzamides and triacetoxybenzamides as one part of target compounds. Deacetylation under acidic conditions gave the other part of our targeted sulfonamide derivatives [28]. The reaction scheme is given in Figure 1.

### 2.3. Biochemical Assays

LPO activities were determined by the procedure of Shindler and Bardsley [29] with slight modification [30,31]. This method is based on the oxidation of chromogenic substrate 2,2′-azino-bis(3-ethylbenzothiazoline-6-sulfonic acid) (ABTS) by hydrogen peroxide (H_2_O_2_), which results in a product which had absorbance at 412 nm [32].

The affinity matrix was synthesised by coupling sulfanilamide as the ligand and l-tyrosine as the spacer arm to CNBr-activated-Sepharose 4B, following the previously published procedure [22] with a slight modification [33,34]. The protein flow in the column eluates was spectrophotometrically determined at 280 nm [35]. All purification steps were performed at 4 °C. The protein quantity was determined at 595 nm according to the Bradford method [36]. Bovine serum albumin was used as the standard protein [37,38,39]. For determination of LPO purity, sodium dodecyl sulphate-polyacrylamide gel electrophoresis (SDS-PAGE) was used according to the procedure of Laemmli [40]. In this application, the imaging method was performed in 10% and 3% acrylamide for the running and the stacking gel, respectively, with 0.1% SDS [41,42,43].

The effects of secondary sulfonamide derivatives (**4a**–**8h**) on LPO purified from fresh bovine milk by affinity chromatography technique were previously determined [22]. In our study, LPO activity was measured in the presence of different concentrations of secondary sulfanilamide derivatives (**4a**–**8h**). A control sample without secondary sulfonamide was taken as 100%. An activity (%)-[Secondary sulfonamide] plot was drawn. For the determination of the inhibition constant (K_i_) values, three different secondary sulfonamide derivative (**4a**–**8h**) concentrations were used. Additionally, ABTS was used as a substrate at five different concentrations. Lineweaver–Burk plots (1/V-1/[ABTS]) were obtained for secondary sulfonamide; the K_i_ and the inhibition type were calculated from these plots [44]. The data obtained were analysed by *t*-test and the results are given as Means ± SD.

## 3. Results and Discussion

Enzymes are biological macromolecules that accelerate or catalyse chemical reactions in biological systems [45,46]. At low concentrations, some chemicals and drugs alter normal enzyme activities by specific enzyme inhibitions [47,48]. On the other hand, sulfonamide derivative drugs were the first systemically used antibiotics and paved the way for the antibiotic revolution in medicine. They were also largely investigated by means of physiological, kinetic, and pharmacological studies [49,50,51]. In addition, these molecules are the most important and largely used zinc binding function for the design of CA isoenzymes inhibitors. It was highlighted that the sulfonamide groups are an ideal ligand for some enzyme active site [52,53].

**Figure 2:**
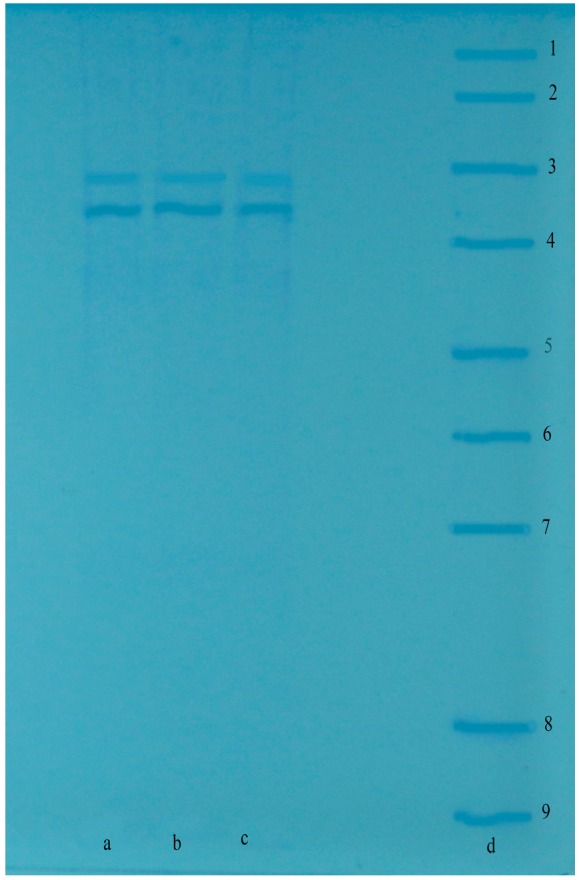
Sodium dodecyl sulphate-polyacrylamide gel electrophoresis (SDS-PAGE) band of LPO. Columns a, b and c are purified LPO from bovine milk by affinity column chromatography. Column d is standard proteins (Line 1: 250 kDa, Line 2: 150 kDa, Line 3: 100 kDa, Line 4: 70 kDa, Line 5: 50 kDa, Line 6: 40 kDa, Line 7: 30 kDa, Line 8: 20 kDa).

Although sulfonamide derivatives are used in therapies, there has been no reported LPO activity for these synthesized compounds (**4a**–**8h**). As show in Table 1, LPO was separately purified from bovine milk by Sepharose 4B-l-tyrosine-sulfonamide affinity chromatography technique. The LPO was purified 407.0-fold with a specific activity of 24.45 EU/mg and overall yield of 75.6% (Table 1). The purification of LPO after Sepharose 4B-l-tyrosine-sulfonamide-affinity chromatography was controlled by SDS-PAGE and a single band was observed for LPO (Figure 2). For secondary sulfonamides (**4a**–**8h**), the inhibitor concentrations causing up to 50% inhibition (IC_50_ values) were determined from the regression analysis graphs. IC_50_ values obtained for LPO are shown in Table 2. From in vitro studies, it was understood that LPO was effectively inhibited by these secondary sulfonamide derivatives (**4a**–**8h**). The inhibition of LPO-catalysed iodination has sometimes been used for designing new antithyroid agents [45]. To understand the nature of LPO inhibition, kinetic experiments with different concentrations of secondary sulfonamides (**4a**–**8h**), varying the respective concentrations of the substrates ABTS for each concentration of the sulfonamide derivatives (**4a**–**8h**) were performed. The Lineweaver–Burk plots were obtained by plotting 1/v versus 1/[ABTS], which showed parallel lines for different concentrations of secondary sulfonamide derivatives (**4a**–**8h**). The lines do not intersect at a common point as shown in Table 2, indicating that secondary sulfonamide derivatives (**4a**–**8h**) inhibition effects against LPO are commonly non-competitive with respect to the ABTS substrates. In this assay, it was suggested that the secondary sulfonamides (**4a**–**8h**) do not directly react with LPO. However, any variation in the concentration of ABTS does not affect the reactivity of secondary sulfonamides (**4a**–**8h**). This is also reflected in the non-competitive nature of the inhibition by the compounds with respect to ABTS. The inhibition data of secondary sulfonamides (**4a**–**8h**) reported here are summarized in Table 2, and the following comments can be drawn from these data. Additionally, LPO inhibition by sulfonamide derivatives (**4a**–**8h**) is dependent on the positioning of the inhibitor in the active site; i.e., the distance between the atoms in the secondary sulfonamide derivatives (**4a**–**8h**) and active site amino acids.

All synthesized secondary sulfonamides derivatives (**4a**–**8h**) exhibited effective inhibitory activity against LPO, one of the prominent enzymes in milk, with a K_i_ values in the range of 1.096 × 10^−3^ ± 0.471 × 10^−3^–1203.83 ± 616.78 µM (Table 2). Additionally, *N*-(sulfathiazole)-3,4,5-triacetoxybenzamide (**6a**) demonstrated the most powerful LPO inhibition effect with low nanomolar K_i_ value (1.096 nM). On the other hand, among the synthesized secondary sulfonamides (**4a**–**8h**) *N*-(sulfadiazine)-*p*-acetoxy-benzamide (**5b**, 260 nM), *N*-(sulfamethazine)-*p*-acetoxybenzamide (**5c**, 80 nM), *N*-(sulfisoxazole) *p*-acetoxybenzamide (**5e**, 934 nM), *N*-(sulfamethizole)-*p*-acetoxybenzamide (**5f**, 324 nM), *N*-(sulfanilamide)-*p*-hydroxybenzamide (**7h**, 217 nM) and *N*-(sulfathiazole)-3,4,5-trihydroxybenzamide (**8a**, 25 nM), showed effective inhibition profiles against bovine milk LPO. Additionally, in general, the acetylated sulfonamides are better inhibitors than the non-acetylated sulfonamides, although the acetylated acids are poorer inhibitors than the non-acetylated acids.

## 4. Conclusions

Secondary sulfonamides incorporating acetoxybenzamide, triacetoxybenzamide, hydroxybenzamide and trihydroxybenzamide–all possessing thiazole, pyrimidine, pyridine, isoxazole and thiadiazole groups–were synthesized, and biological activities were evaluated. Milk is a vital liquid secreted from the mammary glands of females of all mammal species. So, the inhibition of the LPO is very important in terms of the LPO system, which catalyses the oxidation of several different reactions by H_2_O_2_ of a large range of substrates, such as the oxidation of endogenous thiocyanate (SCN^−^) to the antibacterial hypothiocyanite (OSCN^−^). Secondary sulfonamides (**4a**–**8h**) have shown micromolar to nanomolar inhibition against LPO that is vital activity for the innate immune system because of removing bacteria from milk and mucosal secretions. If LPO activity is reduced, this means that the immune system is weakened. This is particularly undesirable, since this affects the immune system of infants that used sulfonamides as a drug.

## Figures and Tables

**Table 1:** Purification of lactoperoxidase (LPO) from bovine milk by Sepharose 4B-l-tyrosine-sulfonamide affinity chromatography.

Purification Steps	Total Volume (mL)	Enzyme Activity (EU/mL)	Total Enzyme Activity (EU/mL·min)	Protein (mg/mL)	Total Protein (mg)	Specific Activity (EU/mg)	Yield (%)	Purification Fold
**Homogenate**	60.0	1.0	60.0	15.0	900.0	0.06	100	1.00
**Sepharose 4B-l-tyrosine-sulfonamide**	10.0	4.5	45.0	0.184	1.84	24.45	75.6	407.0

**Table 2:** The inhibition types, IC_50_ and K_i_ values of some secondary sulfonamide derivatives (**4a**–**8h**) on LPO purified from bovine milk.

No.	Compound Names	IC_50_ (µM)	Ki (µM)	Inhibition Type
**4a**	Sulfathiazole	231.00	38.43 ± 6.06	Non-competitive
**4b**	Sulfadiazine	92.24	90.66 ± 2.52	Non-competitive
**4c**	Sulfamethazine	346.50	198.00 ± 24.46	Non-competitive
**4d**	Sulfapyridine	227.00	65.00 ± 3.61	Competitive
**4e**	Sulfisoxazole	221.70	182.66 ± 34.07	Non-competitive
**4f**	Sulfamethizole	115.50	21.18 ± 5.66	Non-competitive
**4g**	Sulfamerazine	18.73	20.52 ± 2.14	Non-competitive
**4h**	Sulfanilamide	8.48	35.70 ± 4.88	Competitive
**5a**	*N*-(sulfathiazole)-*p*-acetoxybenzamide	99.00	56.06 ± 23.56	Non-competitive
**5b**	*N*-(sulfadiazine)-*p*-acetoxybenzamide	0.015	0.026 ± 0.004	Non-competitive
**5c**	*N*-(sulfamethazine)-*p*-acetoxybenzamide	0.010	0.008 ± 0.003	Competitive
**5d**	*N*-(sulfapyridine)-*p*-acetoxybenzamide	10.04	49.15 ± 19.99	Competitive
**5e**	*N*-(sulfisoxazole)-*p*-acetoxybenzamide	3.03	0.934 ± 0.357	Competitive
**5f**	*N*-(sulfamethizole)-*p*-acetoxybenzamide	0.238	0.324 ± 0.115	Non-competitive
**5g**	*N*-(sulfamerazine)-*p*-acetoxybenzamide	3.94	13.39 ± 6.19	Competitive
**5h**	*N*-(sulfanilamide)-*p*-acetoxybenzamide	3.053	2.29 ± 1.02	Non-competitive
**6a**	*N*-(sulfathiazole)-3,4,5-triacetoxybenzamide	0.656 × 10^−3^	1.096 × 10^−3^ ± 0.471 × 10^−3^	Non-competitive
**6b**	*N*-(sulfadiazine)-3,4,5-triacetoxybenzamide	13.86	2.90 ± 1.13	Competitive
**6c**	*N*-(sulfamethazine)-3,4,5-triacetoxybenzamide	138.60	152.03 ± 48.69	Non-competitive
**6d**	*N*-(sulfapyridine)-3,4,5-triacetoxybenzamide	1.56	1.39 ± 0.15	Non-competitive
**6e**	*N*-(sulfisoxazole)-3,4,5-triacetoxybenzamide	2.03	1.07 ± 0.02	Competitive
**6f**	*N*-(sulfamethizole)-3,4,5-triacetoxybenzamide	6.66	8.55 ± 1.45	Non-competitive
**6g**	*N*-(sulfamerazine)-3,4,5-triacetoxybenzamide	1.28	1.49 ± 0.28	Non-competitive
**6h**	*N*-(sulfanilamide)-3,4,5-triacetoxybenzamide	3.05	2.29 ± 0.98	Non-competitive
**7a**	*N*-(sulfathiazole)-*p*-hydroxybenzamide	15.06	6.85 ± 1.81	Non-competitive
**7b**	*N*-(sulfadiazine)-*p*-hydroxybenzamide	0.040	0.044 ± 0.022	Noncompetitive
**7c**	*N*-(sulfamethazine)-*p*-hydroxybenzamide	0.426	0.484 ± 0.146	Non-competitive
**7d**	*N*-(sulfapyridine)-*p*-hydroxybenzamide	2.96	1.41 ± 0.13	Competitive
**7e**	*N*-(sulfisoxazole)-*p*-hydroxybenzamide	10.83	15.08 ± 3.90	Non-competitive
**7f**	*N*-(sulfamethizole)-*p*-hydroxybenzamide	0.799	0.387 ± 0.150	Competitive
**7g**	*N*-(sulfamerazine)-*p*-hydroxybenzamide	1.759	3.939 ± 1.098	Non-competitive
**7h**	*N*-(sulfanilamide)-*p*-hydroxybenzamide	0.285	0.217 ± 0.099	Non-competitive
**8a**	*N*-(sulfathiazole)-3,4,5-trihydroxybenzamide	0.014	0.025 ± 0.010	Competitive
**8b**	*N*-(sulfadiazine)-3,4,5-trihydroxybenzamide	0.715	1.292 ± 0.465	Non-competitive
**8c**	*N*-(sulfamethazine)-3,4,5-trihydroxybenzamide	2.53	2.17 ± 0.88	Competitive
**8d**	*N*-(sulfapyridine)-3,4,5-trihydroxybenzamide	10.19	5.85 ± 1.28	Competitive
**8e**	*N*-(sulfisoxazole)-3,4,5-trihydroxybenzamide	1.99	1.44 ± 0.52	Competitive
**8f**	*N*-(sulfamethizole)-3,4,5-trihydroxybenzamide	173.25	292.78 ± 61.96	Non-competitive
**8g**	*N*-(sulfamerazine)-3,4,5-trihydroxybenzamide	1.484	3.87 ± 0.43	Non-competitive
**8h**	*N*-(sulfanilamide)-3,4,5-trihydroxybenzamide	1.782	3.104 ± 0.578	Non-competitive
